# Regulation of response to antigen peptides is independent of peptide distribution in lymph node therapeutics

**DOI:** 10.1039/d5bm00328h

**Published:** 2025-08-23

**Authors:** Ryan A. McIlvaine, Senta M. Kapnick, Sean T. Carey, Christopher M. Jewell

**Affiliations:** a Fischell Department of Bioengineering, University of Maryland, College Park 8278 Paint Branch Drive College Park MD 20742 USA; b Robert E. Fischell Institute for Biomedical Devices 8278 Paint Branch Drive College Park MD 20742 USA cmjewell@umd.edu; c Department of Veterans Affairs, VA Maryland Health Care System 10. N Green Street Baltimore MD 21201 USA; d Department of Microbiology and Immunology, University of Maryland Medical School Baltimore MD 21201 USA; e Marlene and Stewart Greenebaum Cancer Center 22 S. Greene Street Suite N9E17 Baltimore MD 21201 USA

## Abstract

Autoimmune disease occurs when immune cells mistakenly identify specific molecules, termed antigens, on healthy cells. There are no cures for these diseases, and existing treatments – including monoclonal antibodies – do not specifically target dysfunctional cells. These challenges have motivated interest in therapies that could achieve antigen-specific immune tolerance. One concept involves co-delivery of self-antigens and regulatory cues to selectively redirect the response to self-antigen. Biomaterials have been investigated in this context owing to the control these technologies offer. We have shown degradable polymer depots encapsulating a self-antigen and delivered to lymph nodes enable reversal of autoimmune paralysis with a single treatment. However, human autoimmune disease is complex and often involves reactivity against sets of autoantigens, highlighting the need to deliver multiple antigens in new immunotherapies. Here we used these depots to encapsulate peptide antigens in distinct loading configuration – either with multiple peptides in a single particle or a single peptide in distinct particles. We show that both formulations are taken up by cells, and drive equivalent T cell responses both *in vitro* and *in vivo*. Notably, when loaded with an immunomodulatory cue, both formulations drive efficient polarization of antigen-specific T cells to regulatory T cells, supporting equivalency of both configurations for tolerizing therapy. Further, MPs can also be loaded with dynamic cargo loading levels without impacting size. The comparability of these two formulations holds significant promise to support simplified mix-and-match design and CMC development of peptide-loaded MPs as a flexible approach for autoimmune therapies.

## Abstract 2

Microparticles are loaded with peptides in distinct configurations, which result in comparable immune cell activity and enable flexible CMC development.

## Graphical abstract

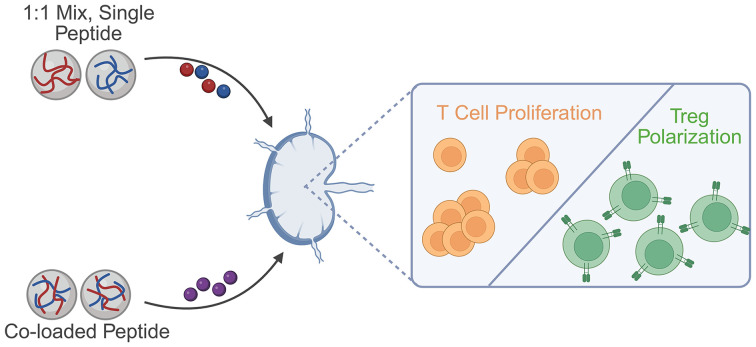

## Introduction

Autoimmune diseases affect up 16% of the global population, with disease prevalence increasing each year.^1,2^ In healthy immunity, immune cells target specific foreign molecules, or antigens, to destroy pathogens without harming other cells. Autoimmunity occurs when immune cells mistakenly target antigens on healthy cells, causing destruction of self-tissue. As there are no cures for these diseases, selective experimental strategies – termed antigen-specific immunotherapies – aim to induce tolerance only to the disease-relevant peptides, preserving normal immune function.

One emerging strategy toward this antigen-specific immune tolerance (ASIT) is the delivery of self-antigen recognized by pathogenic cells during autoimmunity, co-delivered with an immunomodulatory cue, to polarize these cells towards a regulatory phenotype.^3–6^ This technique selectively redirects the immune response towards these specific self-antigens, leaving the remaining healthy immune cells unaffected. While the data is promising, hurdles to translating ASIT to the clinic persist. For example, autoimmune disease in humans often involves T cells reactive to multiple self-antigens, and antigen reactivity can vary from patient to patient.^7^ To combat these challenges, recent clinical trials have begun to investigate the delivery of multiple peptides to induce tolerance in autoimmune diseases such as multiple sclerosis, with more trials ongoing.^8–13^ Thus, the ability and flexibility to deliver multiple antigens could be crucial for successful treatment of autoimmunity in the clinic using this strategy.

Lymph nodes (LNs) are key tissues where signals are coordinated to drive immune responses. Of note, recent clinical trials using direct LN delivery and growing availability of allergy shots administered by direct LN injection demonstrate significant potential.^13–16^ Our lab has developed a platform to induce long-lasting tolerance during autoimmune disease by injecting polymer microparticles (MPs) loaded with self-peptide and tolerizing immune cues directly to LNs. These particles are too large to drain from LNs following injection, instead creating a local depot. These depots release self-antigen and regulatory cues over time, redirecting self-antigen specific cells away from inflammation and toward tolerogenic phenotypes.^17–19^ The encapsulation of cargo in a degradable polymer matrix – formed from poly(lactic-*co*-glycolic acid) (PLGA) – plays a key role in ensuring both cargos are delivered to and retained within LNs. Additionally, this element facilitates protection of cargo, controlled drug release, and co-delivery; the latter is a cardinal feature for many immunotherapies.^20^ However, autoimmune diseases – such as multiple sclerosis (MS) and type 1 diabetes (T1D) – often involve reactivity against several self-antigens. This need to deliver multiple cargos complicates chemistry and manufacturing control (CMC) processes and regulatory strategies for new therapies because of the multi-component nature and the increased complexity of the associated pharmacokinetic (PK) profiles. Thus, formulations that can be synthesized with a single antigen species and then mixed together prior to administration would enable translation of ASIT therapies exploiting LN delivery.

Toward this goal, we used myelin antigens attacked during MS and model antigens – each of which can be tracked using established immunological tools – to test if MPs loaded with multiple antigens are functionally equivalent to simpler formulations in which MPs are formulated with a distinct antigen, then mixed prior to administration. While this concept has been studied for systemic infusion or peripheral injections, this question remains unanswered with respect to local lymph node delivery of immunotherapeutic particles. We show MPs containing multiple peptides, and mixtures of MPs each containing a single peptide, both exhibit similar physicochemical properties and are efficiently internalized by primary antigen presenting cells (APCs). Both formulations also result in antigen-specific T cell proliferation when delivered *via* LN injection. When a regulatory cue is included, the MPs equivalently polarize antigen-specific T cells toward regulatory T cells (T_regs_). Finally, MPs show a much higher peptide loading capacity than tested previously, indicating flexibility in antigen loading and dosing strategies. Taken together, this equivalence informs simplified CMC approaches for ASIT therapies in which particles with defined antigen content can be simply mixed prior to LN injection as a strategy to tolerize against disease-relevant antigen reactivity profiles.

## Materials & methods

### Peptides

Myelin oligodendrocyte glycoprotein, MOG_35–55_ (MEVGWYRSPFSRVVHLYRNGK), FITC fluorescently labelled MOG peptide, ovalbumin peptide OVA_323–339_ (ISQAVHAAHAEINEAGR), and Cy5 fluorescently labeled OVA were purchased from Genscript at >98% purity.

### Synthesis of PLGA microparticles (MPs)

Degradable PLGA (Evonik) MPs were synthesized as previously described^17^*via* a water-in-oil-in-water double emulsion. The inner aqueous phase contained 500 μL of water containing 1 mg of antigen. The organic phase contained 80 mg 50 : 50 DL PLGA with or without two mg Rapa (LC Labs) dissolved in 5 mL dichloromethane (Sigma-Aldrich). To prepare of fluorescently labeled MPs, 4 μL of DiO, DiD, or DiR (Invitrogen) was dissolved with PLGA in the organic phase. Briefly, an initial emulsion was generated by sonicating the inner aqueous phase with the organic phase at 12 W for 30 seconds. This initial emulsion was then homogenized with 40 mL water containing 2% w/v polyvinyl alcohol (Sigma) at 16 000 RPM for three minutes, and was stirred overnight to allow evaporation of dichloromethane. MPs were then filtered through a 40 μm strainer (Corning Falcon) and collected *via* centrifugation at 5000*g* for five minutes at 4 °C. Supernatants were removed, and MPs were washed three times with 500 μL water. MPs were then resuspended at 100 mg mL^−1^ for further use in all studies.

### Characterization of MPs

#### MP characterization

Particle size was determined using an LA-950 laser diffraction analyzer (Horiba). To determine peptide and rapa loading, a known mass of MPs was dissolved in dimethyl-sulfoxide (Sigma-Aldrich). Single peptide concentration was determined using a Micro BCA Protein Assay Kit (Thermo Fisher) according to manufacturer's instructions. For MPs containing multiple, fluorescent peptides, peptide concentration was evaluated using relative fluorescence; fluorescence values were fit to a standard curve of known peptide concentrations to calculate peptide loading per mass of MPs. Both peptide loading methods were found to produce similar results (Table S1).

Cumulative release of peptide from MPs was characterized by suspending aliquots of a known mass of MPs in 1× phosphate buffered saline (PBS) (Corning) and incubating at 37 °C. At 24 hours intervals, an aliquot was removed and centrifuged at 5000*g* for five minutes at 4 °C before removing the supernatant. MP loading was determined as described above to determine the remaining peptide cargo. Peptide release was calculated as a fraction of the peptide loading of each MP batch after synthesis.

Rapa loading was determined *via* UV/Vis spectrophotometry (Thermo Fisher) at 278 nm; absorbance values were fit to a standard curve of known Rapa concentrations to calculate Rapa loading per mass of MPs.

Encapsulation efficiency was calculated by normalizing loading to peptide/drug input during MP synthesis:
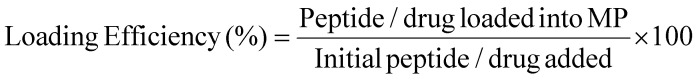


The zeta potential of the MPs in PBS were characterized by a dynamic light scattering (DLS) instrument (Brookhaven Instruments) at 25 °C.

#### MP imaging

Imaging cytometry data was acquired with ImageStreamxMark II (Amnis). MPs were resuspended in PBS at a concentration of 100 μg mL^−1^. At least 10 000 images were collected utilizing a 60× objective. Collected data were analyzed with IDEAS software (Version 6.2, Amnis). Samples were gated for single particles with the area and aspect ratio and for focused particles with the Gradient RMS feature. To determine the fraction of co-loaded MPs containing both MOG- and OVA-labeled peptides, MPs were gated based on median fluorescence intensity using singly loaded particle formulations as internal controls.

Scanning electron microscopy (SEM) images were collected using a Hitachi SU3500 for morphological evaluation of MPs. MPs were dried and sputter coated with 10 μm thick gold–palladium alloy before imaging.

### *In vitro* studies

#### Cell culture media

All cells were cultured in RPMI 1640 + l-Glutamine Media (Thermo Fisher) supplemented with 10% fetal bovine serum (Corning), 2 mM l-glutamine (Gibco), 55 μM β-mercaptoethanol (Sigma-Aldrich), 1× Non-Essential Amino Acids (Fisher Scientific), 10 mM HEPES (Fisher Scientific), and 1× Pen/Strep (Gibco).

#### Flow cytometry

The following antibodies were used for flow cytometry: CD4 (clone GK1.5, diluted 1 : 300), CD11c (clone N418, diluted 1 : 300,) Thy1.1 (clone OX-7, diluted 1 : 500), Thy1.2 (clone 53-2.1, diluted 1 : 500), CD25 (clone PC61, diluted 1 : 200), Foxp3 (clone MF-14, diluted 1 : 100). All antibodies for flow cytometry were purchased from BioLegend. Cells were washed once with PBS containing 1% w/v BSA (FACS buffer) and stained with LIVE/DEAD Fixable Viability Dye (Thermofisher) for 30 minutes at 4 °C. Cells were then washed and blocked with anti CD16/CD32 (BD, Clone 2.4G2, diluted 1 : 25) and stained with surface marker antibodies for 15 minutes at 4 °C. Cells were then washed two times with FACS buffer and were either analyzed immediately or stained for intracellular markers. For intracellular stains, cells were fixed and permeabilized by incubating with Fix/Perm buffer from the Foxp3/Transcription factor staining buffer set (eBioscience) for 30 minutes at 4 °C. Cells were stained with antibodies against intracellular markers for 30 minutes at 4 °C, followed by two washes with Perm/Wash buffer (eBioscience). Cells were analyzed with a CytoFLEX (Beckman Coulter). Flow cytometry data analysis was performed using FlowJo software (Version 10, FlowJo LLC).

#### Bone-marrow derived dendritic cell (BMDCs)

Bone marrow was flushed from the femurs of a C57BL/6J mouse. 10 × 10^6^ cells were cultured in a 100 mm Petri dish in the presence of 20 ng mL^−1^ GM-CSF for six days before harvest for downstream applications.

#### Bone-marrow derived macrophages (BMDMs)

Bone marrow was flushed from the femurs of a C57BL/6J mouse. 15 × 10^6^ cells were cultured in a 100 mm Petri dish in the presence of 50 ng mL^−1^ M-CSF for six days before harvest for downstream applications.

#### Confocal imaging

Eight well microscopy slides (ibidi) were coated with 0.01% w/v poly-l-lysine solution (Sigma-Aldrich) for 30 minutes before drying overnight. For imaging uptake, 400 000 BMDCs were plated per well in 400 μL of culture media with 1 μg mL^−1^ LPS. Imaging slide was cultured for 3–4 hours with MP treatments at 37 °C followed by a gentle PBS wash. Wells were covered in a 2000× dilution of CellMask plasma membrane stain (ThermoFisher) for five minutes at 37 °C, followed by a wash and a 5-minute incubation in 2% paraformaldehyde (Electron Microscopy Sciences) at 37 °C. The slide was washed three times before a 300 nM DAPI stain for five minutes at 37 °C and a final PBS wash. Confocal images were collected using a Zeiss LSM 980 confocal system fitted with an Airyscan 2 module, mounted on an inverted Zeiss Axio Observed Z1 microscope with an oil immersion Plan-Apochromat 63×/1.40 differential interferences contrast (DIC) objective lens (Carl Zeiss, Inc.). Images were acquired in Airyscan super-resolution (SR) mode using a 32-channel GaAsP-PMT Airyscan detector. Zeiss ZEN 2.3 (black) software was used for collection and postprocessing of the images. Airyscan processing was done using an auto-strength 3D method. Post-processing of images was performed in ImageJ 1.53c.

#### Cellular uptake

CD11c dendritic cells were isolated from C57BL/6J mouse splenocytes using positive magnetic selection (Miltenyi) at a purity >95% and plated at 100 000 cells per well in 96-well flat bottom plates. As indicated in the text, certain experiments utilized BMDMs at the same concentrations just described. Following isolation, APCs were stimulated with 1 μg mL^−1^ lipopolysaccharide (LPS) and treated with MPs at indicated concentrations for 24 h. Treatments with mixed MPs utilized MPs with 2× loading, to dose match both cargo and polymer masses (Table S2). MPs were labeled with fluorescent lipophilic dyes, rather than fluorescently labeled cargo, to better visualize populations and to avoid quenching of labeled peptide fluorescence over time.

#### T cell co-cultures

CD11c dendritic cells were isolated and treated as in the dendric cell uptake assay. T cells were then isolated from splenocytes with >95% purity using CD4^+^ negative magnetic selection (StemCell); 300 000 T cells were then co-cultured with MP-treated DCs for three days. For proliferation studies, T cells were labeled with 10 μM CSFE (Invitrogen) or Cell Proliferation Dye eFluor 450 (Invitrogen) prior to culture, and were cultured for three days before flow cytometry staining. To measure cytokine production, cells were cultured for three days before restimulation with Cell Stimulation Cocktail (eBioscience) and Brefeldin A (BioLegend) for 6 hours, following manufacturer's instructions, before flow cytometry staining. For phenotyping studies, cells were cultured for four days before flow cytometry staining.

### *In vivo* studies

#### Ethics

All animal care and research were carried out using protocols approved and overseen by the University of Maryland and the University of Maryland IACUC committee (protocol R-Jan-25-02 in compliance with local, state, and federal guidelines).

#### Animals

All animal care and research was carried out using protocols approved and overseen by the University of Maryland Institutional Animal Care and Use Committee in compliance with local, state, and federal guidelines. C57BL/6J and OT-II (B6.Cg-Tg(TcraTcrb)425Cbn/J) mice were purchased from The Jackson Laboratory. 2D2 (C57BL/6-Tg(Tcra2D2,Tcrb2D2)1Kuch/J), Thy1.1 (B6.PL-Thy1a/CyJ), and 2D2-Thy1.1 mice were bred in University of Maryland facilities. All mice used in these studies were <12 weeks old.

#### Intra-lymph node injections

ILN injection of mice was performed as previously described.^17,18^ Hair surrounding the injection site was removed using a mild depilatory cream and mice were injected subcutaneously at the tail base with 10 μL Evans blue (Sigma-Aldrich). Three hours after dye injection, both inguinal LNs were identified and injected with 1 mg of MPs. MPs were suspended in 10 μL sterile PBS and injected using a 31 g insulin syringe.

#### *In vivo* imaging

Animals were injected iLN with fluorescently labelled MPs as described above. One and three days following injection, mice were imaged under anesthesia using an IVIS Spectrum *in vivo* imaging system (PerkinElmer), before mice were euthanized and LNs were surgically excised and imaged. Exposure and lamp settings were determined using untreated control. Image analysis was performed using Living Image Software (Version 4.7.4, PerkinElmer).

#### Adoptive transfer studies

C57BL/6J or C57BL/6J-Thy1.1 mice were immunized iLN with MP treatments. One day following iLN treatment, CD4^+^ T cells were isolated from splenocytes of 2D2 or OT-II mice using CD4 negative bead-based magnetic selection (Stemcell) per the manufacturer's instructions. 2 × 10^6^ 2D2 and 2 × 10^6^ OT-II CD4^+^ T cells were adoptively transferred to host mice *via* intravenous injection. In proliferation studies, T cells were labeled with CSFE or eFluor 450 prior to adoptive transfer. At indicated times following T cell transfer, treated mice were sacrificed and LNs were processed and analyzed by flow cytometry. Transferred cells were identified by proliferative dye, Thy1.1 and/or Thy1.2 staining.

### Statistical analysis and reproducibility

Analyses were carried out with GraphPad Prism (version 10.3.0). The specifics of statistical tests for each experiment and the number of replicates are detailed in figure legends. Error bars in all panels represent the mean ± standard error and *p* values ≤0.05 were considered significant with levels of significance defined as *p < 0.05, **p < 0.01, ***p < 0.001, ****p < 0.0001. For comparisons of more than two groups, one-way ANOVA with Tukey's post-test was performed. For comparisons of two groups, two-tailed Welch's *t*-test was performed.

## Results

### MPs efficiently encapsulate multiple peptide antigens

**Fig. 1:**
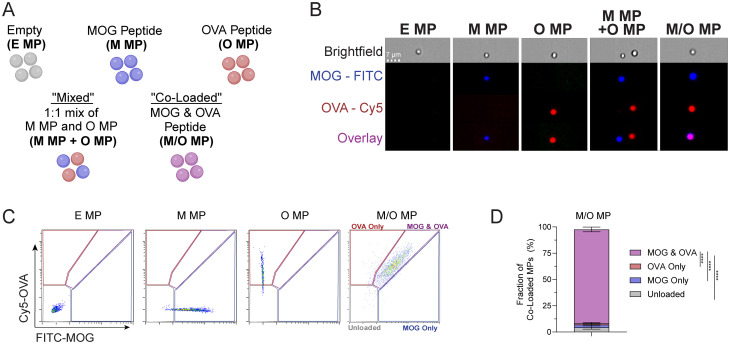
MOG and OVA peptides can be loaded into a single MP. (A) MP formulation naming conventions. (B) Representative imaging cytometer data of fluorescent peptide cargo for individual MP formulations. Scale bar: 7 μm. (C) Representative gating strategy on imaging cytometer to determine co-loading efficiency of peptide cargo. (D) Quantification of peptide co-loading efficiency using imaging cytometry. Data is representative of three or more experiments. ****P < 0.0001 determined with one-way ANOVA and Tukey *post hoc* multiple comparisons.

We hypothesized that multiple peptide antigens could be loaded into polymer MPs at a dose similar to individually loaded MPs. To test this, we first synthesized MPs containing myelin oligodendrocyte glycoprotein 35–55 (MOG) and ovalbumin 323–339 (OVA) peptides. These peptides were formulated in MP depots in two configurations, either (i) co-loaded in the same particle (MOG and OVA), or (ii) loaded separately with one type of peptide (MOG or OVA) per particle (Fig. 1A). These formulations were designated: (i) empty (E MP), (ii) MOG MPs (M MP), (iii) OVA MPs (O MP), (iv) mixed MPs (M MP + O MP), and (v) co-loaded MPs (M/O MP). Peptide-loading measurements using BCA assays confirmed that M MPs, O MPs, and M/O MPs can be loaded with peptide at a similar level and efficiency; further, M/O MPs have comparable release profiles compared to M MPs and O MPs (Table 1 and Fig. S1). Laser diffraction analysis of particle size confirmed the formulations exhibited comparable sizes when containing individual or multiple peptides (Fig. S2). Zeta potential measurements indicate each formulation is approximately neutral (Table 1). Scanning electron microscopy also demonstrates that MPs have a similar smooth topography across all formulations (Fig. S3).

**Table 1:** Size and loading of MP formulations

Formulation	Size (μm)	MOG (μg mg^−1^ MP)	MOG loading efficiency (%)	OVA (μg mg^−1^ MP)	OVA loading efficiency (%)	Zeta potential (mV)
E MP	3.11 ± 0.02	—	—	—	—	2.92 ± 3.92
M MP	3.38 ± 0.06	7.88 ± 0.19	38.5 ± 2.3	—	—	0.26 ± 1.46
O MP	3.87 ± 0.05	—	—	5.83 ± 0.96	23.6 ± 2.9	2.73 ± 1.15
M/O MP	3.59 ± 0.06	6.73 ± 1.13	30.9 ± 4.4	4.03 ± 0.73	18.8 ± 1.7	4.21 ± 0.39

While the study above quantified bulk loading levels for each MP configuration, the data do not reveal whether cargo is truly co-loaded in each individual particle, differentiating co-loaded MPs from mixed MPs. Thus, we next used imaging cytometry and peptides labeled with unique fluorescent dyes (*i.e.*, MOG-FITC and OVA-Cy5) to assess cargo distribution and co-loading on a per particle basis. Particle size across loading configurations was consistent with laser diffraction analysis and fluorescently-labeled cargo was readily distinguished, confirming the distinct cargo loading schemes (Fig. 1B and Fig. S4). Quantification of particle populations using gating (Fig. 1C) revealed that more than 90% of M/O MPs particles were loaded with both peptides (Fig. 1D). These data indicate particles fabricated with one or multiple peptides exhibit similar product profiles, including cargo size and loading.

### *In vitro* dendritic cell uptake is unaffected by MP cargo

**Fig. 2:**
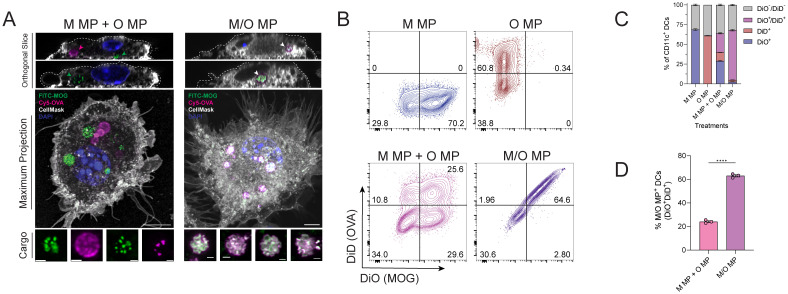
Mixed and co-loaded MPs are internalized by BMDCs. (A) Representative confocal microscopy images of BMDCs. Orthogonal slices (top) are taken from the maximum projection (middle) to show internalization. Scale bar = 5 μm. Fluorescently-labelled MP cargo from intracellular compartments is shown (bottom). Scale bar = 1 μm. (B) Representative flow plots illustrating MP among CD11c^+^ DCs. (C) Quantification of frequency of DCs without MPs (no fluorescent signal) or with MOG MPs (DiO^+^), OVA MPs (DiD^+^), mixed MPs (DiO^+^/DiD^+^) or co-loaded MPs (DiO^+^/DiD^+^). (D). Quantification of DC frequency that contains MPs with both peptides. Data is representative of two or more experiments. ****P < 0.0001 determined with one-way ANOVA and Tukey *post hoc* multiple comparisons, or two-tailed Welch's *t*-test.

To test if distinct MP formulations impact uptake by APCs, pan-dendritic cells (DCs) were cultured from bone marrow and treated with MPs singly loaded with fluorescently-labeled MOG (M MP) or OVA (O MP), mixed MPs (M MP + O MP), or co-loaded MPs (M/O MPs) and imaged using confocal microscopy (Fig. 2A and Fig. S5). Orthogonal optical sections of individual cells confirmed internalization by individual cells for both mixed and co-loaded MP cargo. Imaging also revealed internalization of distinct punctate areas of labeled cargo, demonstrating these cells internalize more than one particle. Cargo identified in DCs treated with mixed MPs (M MP + O MP) exhibited spatial separation between the labeled peptides. In contrast, fluorescently-labeled cargo observed in the cargo cells treated with co-loaded MP (M/O MP) was observed in overlapping regions, highlighting different intra-cellular distributions between the two MP formulations.

To quantify whether peptide loading configurations result in differential uptake levels by DCs, we labeled MPs during assembly with lipophilic dyes for tracking. CD11c^+^ mouse splenic DCs were treated with either fluorescently-labeled single-peptide MPs (M MP-DiO or O MP-DiD), mixed MPs (M MP-DiO + O MP-DiD), co-loaded MPs (M/O MP-DiO/DiD), soluble fluorescent peptide (Fig. S6), or unlabeled MPs; the latter served as a negative control for fluorescence (Fig. S7). Quantification using flow cytometry revealed that approximately 65% of DCs internalized particles, regardless of formulation (Fig. 2B and C). Notably, only 25% of DCs treated with the 1 : 1 mix (M MP-DiO + O MP-DiD) internalized both peptides compared with 80% of DCs treated with MPs co-encapsulating two peptides (M/O MP-DiO/DiD) (Fig. 2D). Macrophages are highly phagocytic immune cells that can also act as APCs. Thus, macrophages were cultured from bone marrow and also treated with MPs as above. Similar trends were also observed in this cell type, with M/O MPs better delivering two peptides to a single cell (Fig. S8). Thus, while delivery of two peptides to a single cell is better achieved by co-loaded MPs, overall uptake of MPs was unaffected by peptide cargo content.

### T cells divide equivalently *in vitro* in response to mixed and co-loaded MP treatments

**Fig. 3:**
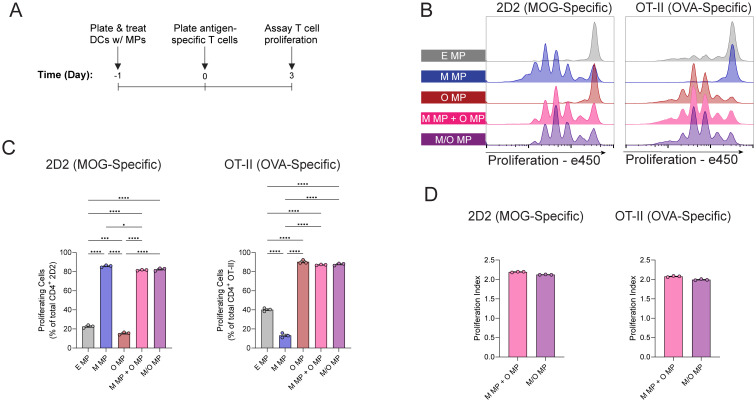
Mixed and co-loaded MPs equivalently drive T cell proliferation *in vitro*. (A) Schematic of experimental approach. (B) Representative histograms of proliferation among 2D2 (MOG-specific, left panel) & OT-II (OVA-specific, right panel) CD4 T cells. (C) Quantification of the frequency of proliferated cells for each treatment. (D) Proliferation index of 2D2 and OT-II T cells for mixed and co-loaded MP-treated groups. Data is representative of three experiments; *P < 0.05, ***P < 0.001, ****P < 0.0001 determined with one-way ANOVA and Tukey *post hoc* multiple comparisons, or two-tailed Welch's *t*-test.

Our data indicate improved co-localization of peptide in DCs using co-loaded MPs. However, a streamlined CMC process would be facilitated using particles encapsulating individual peptides that can be mixed prior to injection, eliminating additional process development steps. Thus, we investigated whether differences in uptake translate to a difference in T cell expansion. To test this, splenic DCs isolated from congenic (Thy1.1) mice were treated with MPs as described above, followed by co-culture with CD4^+^ T cells isolated from 2D2-Thy1.1 or OT-II-Thy1.2 transgenic mice; the T cell receptors in these mice specifically recognize either MOG (2D2) or OVA (OT-II) peptides (Fig. 3A). After three days, division among CD4 T cells was evaluated using flow cytometry. Congenic markers were used to identify 2D2 and OT-II T cell populations in co-cultures (Fig. S9) in combination with proliferation dyes, where each peak represents a generation of cell division (Fig. 3B). As expected, cells treated with MPs without their cognate antigen showed diminished viability (Fig. S10). Particles containing the cognate antigen drive division of T cells with corresponding reactivity: M MPs drove division of 2D2 T cells but not OT-II T cells in culture (Fig. 3C, left panel), while OT-II T cells divided in response to O MPs but not M MP treatment (Fig. 3C, right panel). Interestingly, there was no significant difference in the frequency of proliferating MOG-specific (2D2) or OVA-specific (OT-II) T cells in response to co-loaded (M/O MP) compared with mixed (M MP + O MP) treatments (Fig. 3C). This result was observed despite differences in the frequency of DiO^+^/DiD^+^ DCs indicated by uptake studies. To further examine whether the peptide co-delivery strategy (co-loaded *vs.* mixed) impacted the extent of division among proliferating cells, the proliferation index (PI) was calculated. This metric, calculated by measuring the average number of divisions undergone by all cells, better captures the difference between two proliferating cell populations. No significant difference in the PI was observed (Fig. 3D), indicating equivalence between the mixed and co-loaded MP formulations with respect to their ability to induce antigen-specific T cell proliferation *in vitro*. We also investigated the functionality of these antigen-specific divided T cells by performing a restimulation of MP treated cells. Consistent with priming, these cells produced IFN-γ in response to restimulation, with peptide-loaded MPs producing significantly more cytokine relative to the co-cultures of DCs treated with empty MPs or untreated cells (Fig. S11).

### Mixed and co-loaded MPs drive equivalent antigen-specific T cell division in mice

**Fig. 4:**
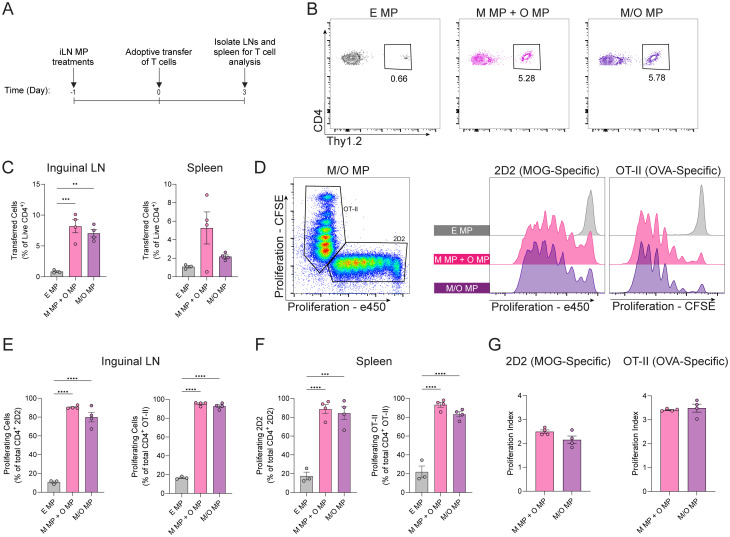
Mixed and co-loaded MPs equivalently drive T cell proliferation *in vivo*. (A) Schematic of experimental approach. (B) Representative flow plot of transferred cell recovery and (C) quantification. (D) Representative flow plot of 2D2 *vs.* OT-II gating and proliferation histograms. (E) Quantification of frequency of proliferated cells among transferred cells recovered from inguinal LNs. (F) Quantification of frequency of proliferated cells among transferred cells recovered from spleen. (G) Proliferation index for 2D2 & OT-II cells in the inguinal LN. *n* = 4; **P < 0.01, ***P < 0.001, ****P < 0.0001 determined with one-way ANOVA and Tukey *post hoc* multiple comparisons, or two-tailed Welch's *t*-test.

To test if MP treatments drive equivalent proliferation of antigen-specific T cells in mice, we treated inguinal lymph nodes (LNs) with co-loaded or mixed MPs, or empty MPs as a control. Direct iLN injection using diffusion-limited (*i.e.* size) particles allow for targeted delivery and retention of MP-loaded cargo in LN; these particles are too large to drain out of the LN, and instead degrade to slowly release cargo in the node (Fig. S12).^21^ 2D2 (MOG-specific) and OT-II (OVA-specific) CD4^+^ T cells, both isolated from Thy1.2 transgenic mice, were labeled with proliferation dyes exhibiting distinct spectra. This approach enables tracking of cell division between the cell populations. One day after mice were treated with MPs, a 1 : 1 mixture of the labeled antigen-specific T cells was infused into mice to ensure a robust pool of each antigen-specific cell population. After three days, inguinal, axillary, brachial LNs and spleens were isolated from treated mice and evaluated *via* flow cytometry (Fig. 4A). In these studies, a significantly higher frequency of antigen-specific, transferred cells were recovered from mice treated with either antigen-containing MP formulations, relative to EMP (Fig. 4B and Fig. S13). Both 2D2 (MOG-specific) and OT-II (OVA-specific) CD4^+^ T cells recovered from LNs divided in response to co-loaded and mixed MP treatments (Fig. 4C). Importantly, no quantifiable differences were observed in the frequency of proliferating cells (Fig. 4D and E) or PI (Fig. 4F) among antigen-specific donor cells recovered from either co-loaded or mixed MP-treated mice. A similar trend was seen in the axillary and brachial LNs (Fig. S14). Thus, peptide – whether co-loaded into single MPs or delivered *via* a mixed MP strategy, results in equivalent recruitment and expansion of antigen-specific T cells to immune tissues in mice.

### Rapamycin-loaded mixed and co-loaded MPs similarly drive T_reg_ phenotype

**Fig. 5:**
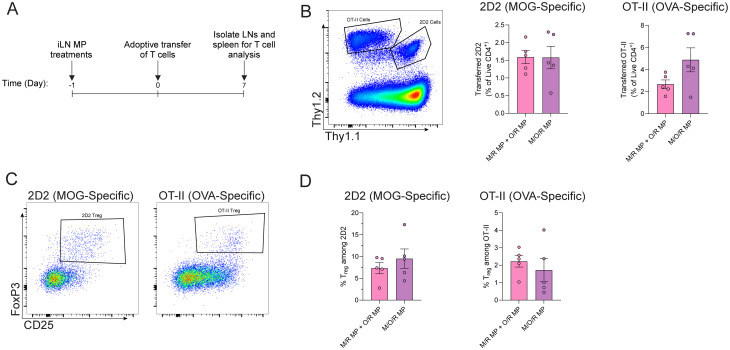
Mixed and co-loaded MPs loaded with rapamycin drive Treg populations *in vivo*. (A) Schematic of experimental approach. (B) Representative flow plot of identification of transferred cells (left); quantification of the frequency of 2D2 (left graph) or OT-II (right graph) CD4 T cells. (C) Representative flow plots of T_reg_ gating for 2D2 and OT-II cells. (D) Quantification of the frequency of T_regs_ among 2D2 and OT-II cells recovered from inguinal LN. *n* = 5; significance determined with two-tailed Welch's *t*-test.

Prior work from our lab has demonstrated that MPs delivering antigen alongside the immunomodulatory cue – rapamycin (rapa) – drives efficacy in multiple pre-clinical models of autoimmune disease.^17,18^ Treatment in these models is antigen-specific and results in a shift of T cell phenotypes towards regulatory T cells (T_reg_) that play a key role in efficacy. Therefore, we next tested if co-loaded or mixed approaches to multiple peptide delivery in combination with rapa polarize antigen-specific T cell to T_reg_. To this end, mixed (M/R MP + O/R MP) or co-loaded (M/O/R MPs) peptide MPs were synthesized with the addition of rapa in the organic phase, as described previously (Table 2). The addition of rapa had no impact on MP size or peptide loading (Table 2). As seen above in Fig. 4, iLN treatment with empty MPs did not lead to cell expansion, and so mice were only treated with mixed or co-loaded MPs and T cells were adoptively transferred as above. After seven days, inguinal LNs and spleens were isolated, processed, and stained for T_regs_ (CD25^+^FoxP3^+^) among 2D2 (Thy1.1^+^Thy1.2^+^) and OT-II (Thy1.1^−^Thy1.2^+^) CD4 T cells (Fig. 5A and Fig. S15).

**Table 2:** Size and loading of rapa MP formulations

Formulation	Size (μm)	MOG (μg mg^−1^ MP)	MOG loading efficiency (%)	OVA (μg mg^−1^ MP)	OVA loading efficiency (%)	Rapa (μg mg^−1^ MP)	Rapa loading efficiency (%)	Zeta potential (mV)
M/R MP	3.26 ± 0.06	7.66 ± 0.89	35.7 ± 5.4	—	—	15.20 ± 0.09	35.2 ± 1.8	−3.06 ± 0.49
O/R MP	3.20 ± 0.03	—	—	4.98 ± 0.46	21.7 ± 2.7	16.39 ± 1.62	35.4 ± 2.9	−2.41 ± 1.09
M/O/R MP	3.48 ± 0.03	7.32 ± 0.42	28.1 ± 2.6	4.78 ± 0.73	18.6 ± 3.8	15.04 ± 2.07	28.4 ± 2.6	0.75 ± 7.45

An equivalent proportion of 2D2 (MOG-specific) and OT-II (OVA-specific) CD4 T cells were recovered from MP-treated mice (Fig. 5B). Importantly, no significant difference in the frequency of T_regs_ was observed among the recovered antigen-specific cells exhibiting specificity for either MOG or OVA (Fig. 5C and D). These data demonstrate the delivery of immunomodulatory cues, such as rapa, alongside one or multiple peptides can equivalently alter T cell phenotype, regardless the formulation scheme.

### MPs can be flexibly loaded without impacting material properties

**Fig. 6:**
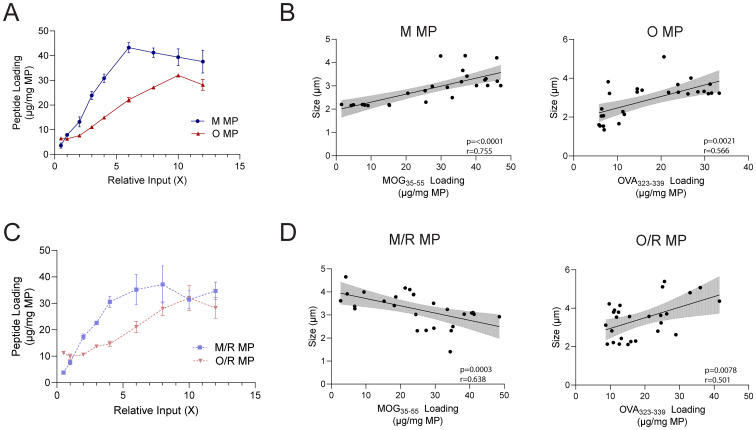
MPs can be loaded with greater amounts of peptide with limited changes to MP size. (A) Loading curve of peptide in M and O MPs. (B) Correlation between size of MPs and peptide loading in M and O MPs. (C) Loading curve of peptide in M/R and O/R MPs. (D) Correlation between size of MPs and peptide loading in M/R and O/R MPs. 95% confidence intervals shown as shaded regions.

Having established the equivalence above, we tested the ability of MPs to encapsulate increasing amounts of antigen or immunomodulatory cue to support pre-clinical development and future dose-escalation. Initially peptide-only MPs were synthesized by increasing the peptide input during assembly in a stepwise fashion, up to 12-fold compared to the formulations used prior. The loading of peptide in M MPs and O MPs was examined – rather than M/O MPs – to determine the maximum potential loading without interference between peptide cargos. These studies revealed a single-peptide MP could be loaded with up to a 5-fold increase in peptide compared to the MP peptide dose used in the previous studies (Fig. 6A) – for MOG, up to 40 μg mg^−1^ MP was loaded before capacity plateaued. All particles fell within 2–5 μm in size, with size trending upwards as peptide loading increases (Fig. 6B). We also tested if the loading of an immunomodulatory cue, such as rapa, would impact the maximum peptide capacity of peptide in MPs. M/R and O/R MPs were fabricated in a stepwise fashion as above, holding rapa input constant during synthesis. Similarly, these MPs could be loaded with an approximately 5-fold increase in peptide (Fig. 6C), without a reduction in rapa as peptide loading increased (Fig. S16). These MPs also exhibited sizes in the range of 2–5 μm. Similar to M MPs and O MPs, O/R MPs trended upward in size as loading increased (Fig. 6D). Interestingly, M/R MPs exhibited the opposite trend – decreasing in size as peptide loading increased, which may be due to unique interactions between rapa and MOG peptide.

Lastly, we sought to determine the maximum loading of rapa, independent of peptide loading. To test this, rapa-only MPs were synthesized with stepwise increases of rapa input. The MPs were found to have a maximum drug loading approximately 7 times as large as the standard formulation (Fig. S17). These MPs were also within 2–4 μm in size, without notable trends as rapa loading increased (Fig. S18). Taken together, these data suggest the platform is flexible and can accommodate a range of cargo.

## Discussion

The delivery of peptide antigens as part of novel, antigen-specific immunotherapies has shown promise in a number pre-clinical applications.^22–24^ However, human autoimmune disease is complex and involves a family of autoantigens, highlighting the need to deliver multiple antigens in new immunotherapies. Despite promising pre-clinical data, many clinical trials that deliver several peptides utilize soluble delivery, resulting in cargo that is quickly cleared from the body.^25^ Building on our past work, we show intra-lymph node depots can be formulated with one or multiple antigens without hindering tolerogenic function. Our data indicate that peptide delivery using mixtures of individually-loaded MPs elicits equivalent immunological outcomes (*e.g.*, proliferation, T_reg_ polarization) compared with co-loaded MP formulations. This finding holds promise for clinical translation, as using mixtures of singly-loaded MPs to deliver multiple peptides facilitates a simpler design and a more efficient regulatory pathway. For instance, during process development, the challenges associated with consistently loading and verifying multiple peptides within a single particle are eliminated. Instead, MPs individually loaded with single peptides could be manufactured and combined during fill-finish to create the final product. This off-the-shelf approach would maximize accessibility. In the future, this may also allow patient-specific mixtures to be formulated for personalized antigen selection. Although the data presented here support the functional equivalence of co-loaded and mixed MPs in modulating immune cells, one risk that requires further study is scenarios in which certain immunomodulatory cargos may benefit from being co-delivered to the same cell (*i.e.*, in a single particle). Overall, the equivalence of these two MP formulations further supports the flexible use of either to develop desired immunotherapies.

Here, MPs are injected directly into LNs and are designed to be too large to drain out *via* lymphatics.^18^ This injection method allows for the direct delivery of MP cargo to immune cells in the LN, without loss due to systemic delivery. Recent studies show that iLN MP treatments are safe in non-human primates while additional clinical trials examining iLN delivery of immunotherapies for allergy and autoimmune disease are also underway.^19,26,27^ Notably, these trials target the same inguinal lymph nodes as seen in the *in vivo* studies described above. Further, patients seeking allergy relief can now obtain immunotherapies that utilize iLN injections at some university medical centers, highlighting the translational potential of this therapeutic route.^28,29^

While we investigate only two peptides in this study, our data suggests that we can load a much greater dose of peptide or number of peptides using the same synthesis method. In addition, we observed little reduction in loading efficiency when two peptides are combined during assembly (co-loaded MPs). However, the loading efficiency of peptides can differ due to their overall size, charge, or hydrophobicity.^30^ The ability to modulate peptide dose by increasing input during synthesis can overcome these loading limitations, allowing for control of peptide dose in each MP formulation. Recent clinical trials have tested the delivery of a pool of peptide antigens in experimental tolerancing strategies.^8,9^ These trials highlight likely candidates for MP peptide delivery to treat human autoimmunity. A formulation of mixed particles, each containing one of these peptides, could be defined for the drug candidate to test clinically. As described above, a persistent challenge in ASIT is the selection of autoantigen(s) to deliver; the ability of this particle platform to deliver multiple antigens eases this burden, which could improve the likelihood of successful translation of antigen-specific therapies in the clinic. To further support clinical translation, scalable and GMP-compatible fabrication methods are critical. Although the MPs in this study were synthesized using a water-in-oil-in-water double emulsion method, alternative approaches such as spray-drying and electrospraying have been explored.^31,32^ These methods offer advantages for industrial-scale production, including improved control over particle characteristics and compatibility with GMP manufacturing standards. Together, the modularity of the MP platform and the adaptability of synthesis methods underscore its potential for developing clinically relevant antigen-specific therapies.

## Conclusion

New ASIT therapies for autoimmune disease will likely require tolerance against multiple self-antigens. The work here demonstrates a combination of singly loaded MPs, and MPs that load both peptides within a single particle, can effectively drive T cell proliferation and, when loaded with an immunomodulatory cue, cell phenotype *in vitro* and *in vivo*. This equivalence could support simplified design and CMC development of peptide-loaded MPs as a flexible approach for autoimmune therapies.

## Author contributions

Conceptualization: R. A. M., C. M. J. Investigation: R. A. M., S. M. K., S. T. C. Visualization: R. A. M., S. M. K. Writing: R. A. M., S. M. K., S. T. C., C. M. J. Supervision: C. M. J.

## Conflicts of interest

The authors declare the following competing financial interest(s): CMJ and SMK are employees of the VA Maryland Healthcare System. The views in this paper do not reflect the views of the Department of Veterans Affairs or the United States Government. CMJ has equity positions in Cartesian Therapeutics, Nodal Therapeutics, and Barinthus Biotherapeutics. The authors declare that they have no other known competing financial interests or personal relationships that could have appeared to influence the work reported in this paper.

## Supplementary Material

BM-013-D5BM00328H-s001

## Data Availability

The authors confirm that the data supporting the findings of this study are available within the article and its SI. Supplementary information: Additional MP characterization, soluble controls, flow cytometry gating schemes and viability, IVIS imaging, cytokine restimulation assay, and supplementary images. See DOI: https://doi.org/10.1039/d5bm00328h.
