# Supplementary material for: Regulation of response to antigen peptides is independent of peptide distribution in lymph node therapeutics
Source: Biomater Sci. 2025 Aug 23;13(19):5538–49. doi: 10.1039/d5bm00328h (PMC12394929; doi:10.1039/d5bm00328h)
Supplement: BM-013-D5BM00328H-s001 [file BM-013-D5BM00328H-s001.pdf]

Supplementary Information (SI) for Biomaterials Science.
This journal is © The Royal Society of Chemistry 2025

SUPPORTING INFORMATION

Regulation of response to antigen peptides is independent of peptide distribution in lymph node therapeutics

*Ryan A. McIlvaine[1,2], Senta M. Kapnick[2,3], Sean T. Carey[1,2], and Christopher M. Jewell[2,3,4,5]**

[1]Fischell Department of Bioengineering, University of Maryland, College Park, 8278 Paint Branch Drive, College Park, MD 20742, USA

[2]Robert E. Fischell Institute for Biomedical Devices, 8278 Paint Branch Drive, College Park, MD 20742, USA

[3]Department of Veterans Affairs, VA Maryland Health Care System, 10. N Green Street, Baltimore, MD 21201, USA

[4]Department of Microbiology and Immunology, University of Maryland Medical School, Baltimore, MD, 21201, USA

[5]Marlene and Stewart Greenebaum Cancer Center, 22 S. Greene Street, Suite N9E17, Baltimore, MD, 21201, USA

*Corresponding author

SUPPLEMENTAL TABLES

Table S1. MOG peptide loading as determined by BCA and fluorescence of MOG and FITC-MOG MPs.

Formulation	MOG (μg/mg) via BCA	MOG (μg/mg) via Fluorescence
MOG MP	7.88 ± 0.19	-
FITC-MOG MP	7.24 ± 0.67	7.25 ± 0.66

Table S2. Peptide loading of MPs with 2X peptide loading for *in vitro* dose-matching.

Formulation	Size (μm)	MOG (μg/mg)	OVA (μg/mg)	Rapa (μg/mg)
M MP (2X Input)	2.17 ± 0.01	13.17 ± 2.02	-	-
O MP (2X Input)	2.86 ± 0.68	-	7.64 ± 0.42	-
M/R MP (2X Input)	3.60 ± 0.11	17.37 ± 1.07	-	22.73 ± 1.50
O/R MP (2X Input)	3.97 ± 0.13	-	10.62 ± 0.47	14.83 ± 1.78

SUPPLEMENTAL FIGURES

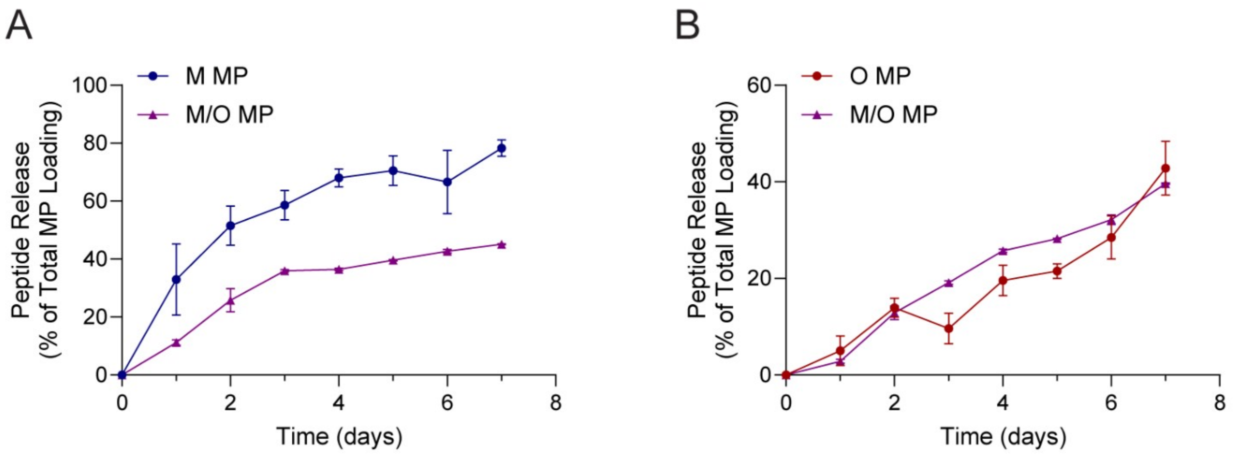

Figure S1. Peptide release profiles of M MPs, O MPs, and M/O MPs over 7 days. (A) MOG peptide release from M MPs and M/O MPs. (B) OVA peptide release from O MPs and M/O MPs.

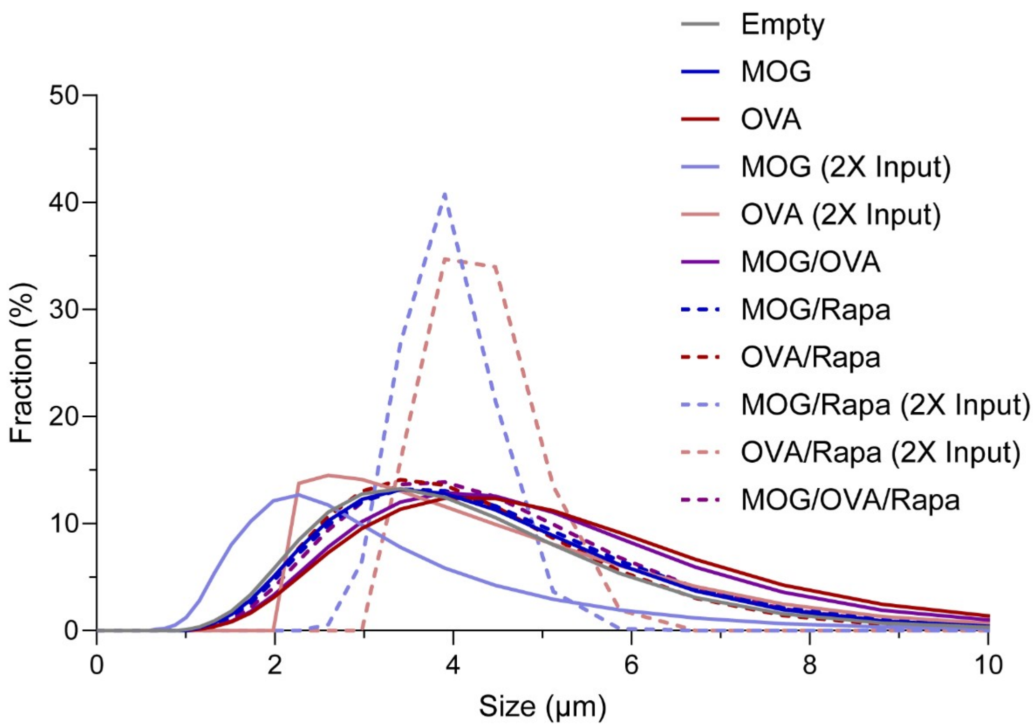

Figure S2. Laser scattering size distribution curves for all MP formulations.

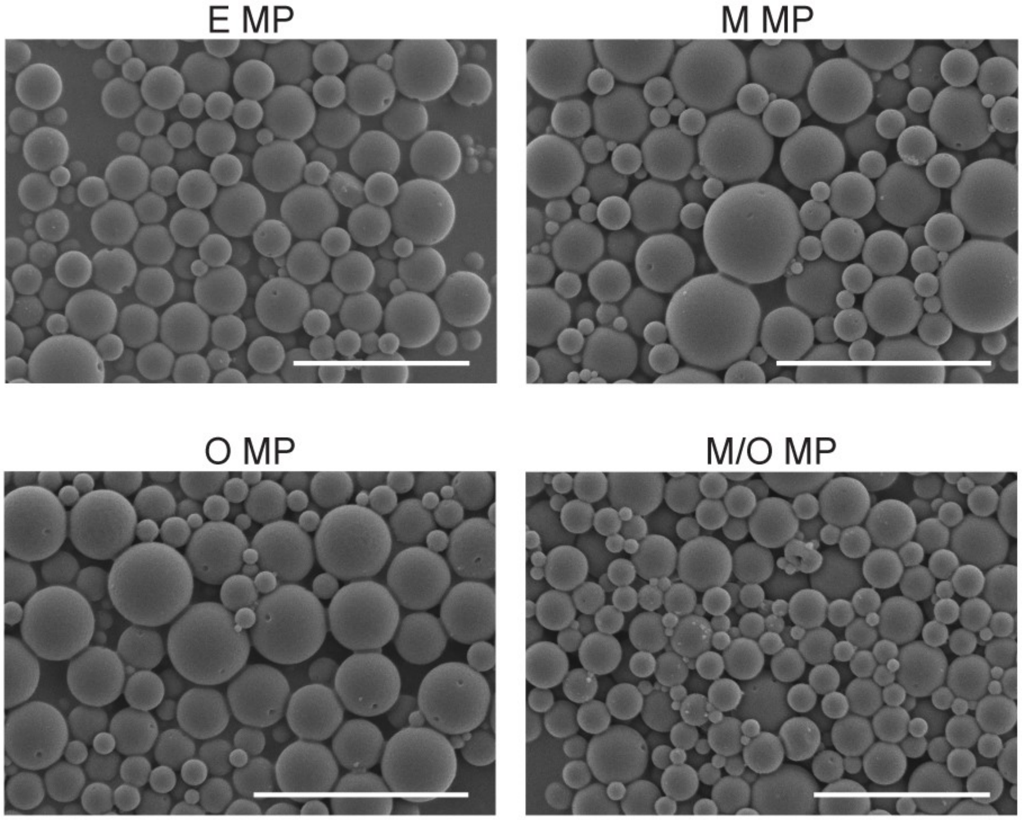

Figure S3. Representative SEM images of MP formulations. Scale bar = 10 μm.

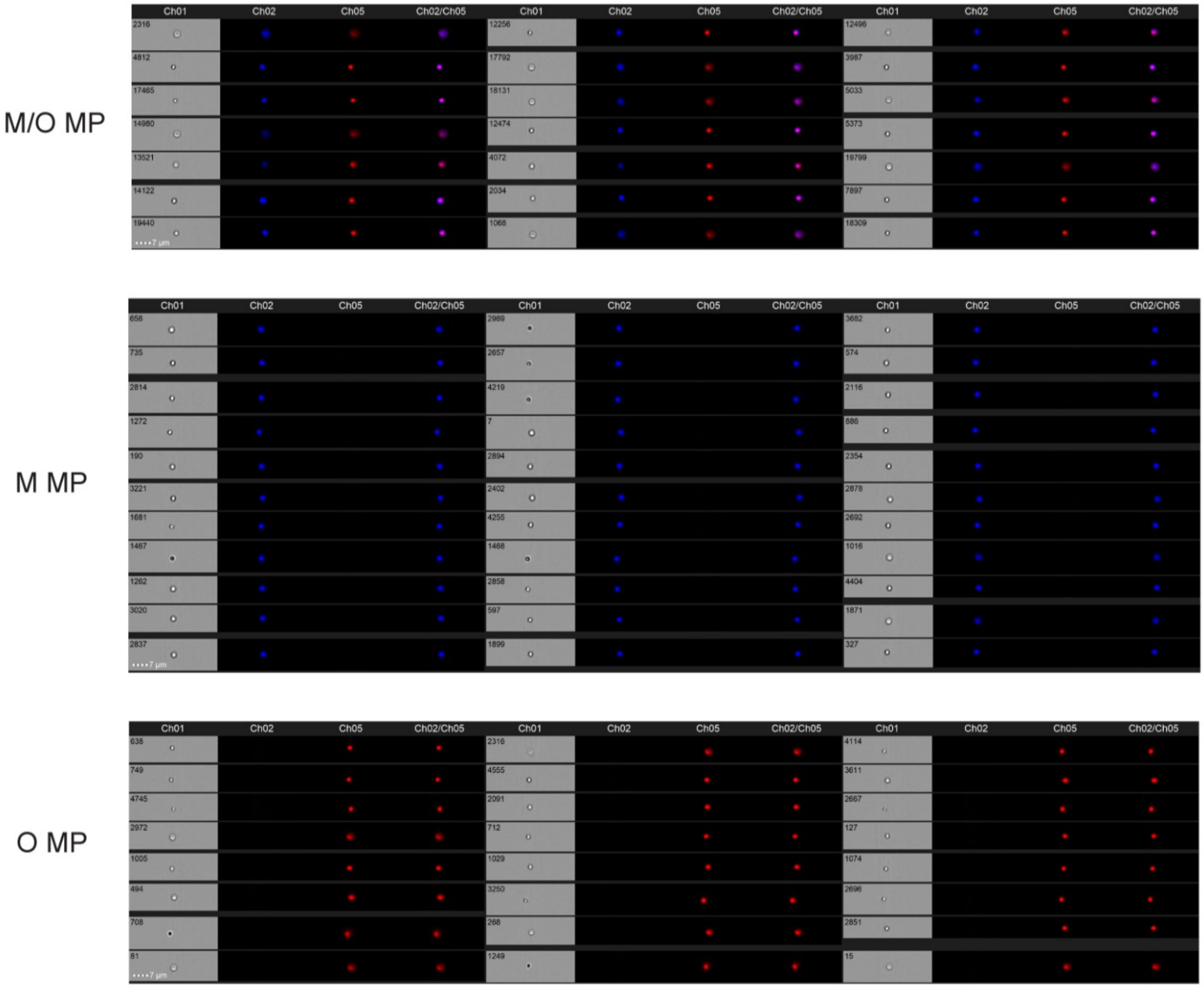

Figure S4. Representative imaging cytometer images for M/O MPs, M MPs, and O MPs.

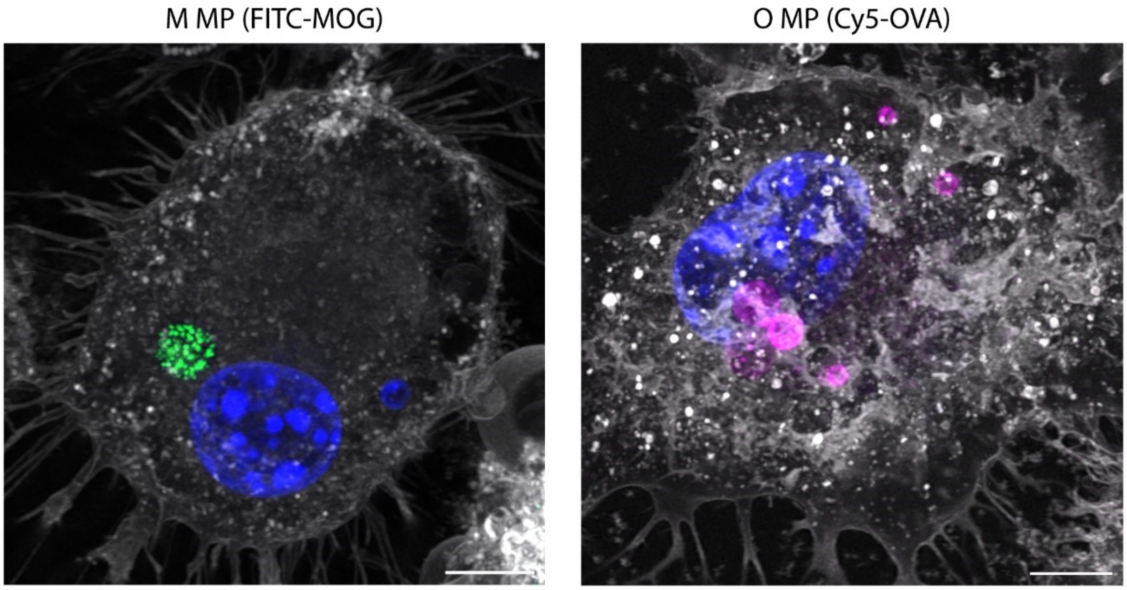

Figure S5. Representative microscopy images for M MP and O MP treated BMDCs. Scale bar = 5 µm.

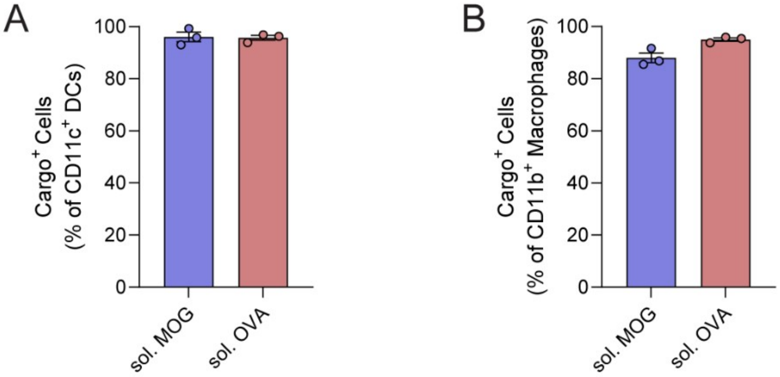

Figure S6. Soluble peptide controls in flow cytometry experiments for (A) CD11c+ DCs and (B) CD11b+ macrophages. Data is representative of two or more experiments.

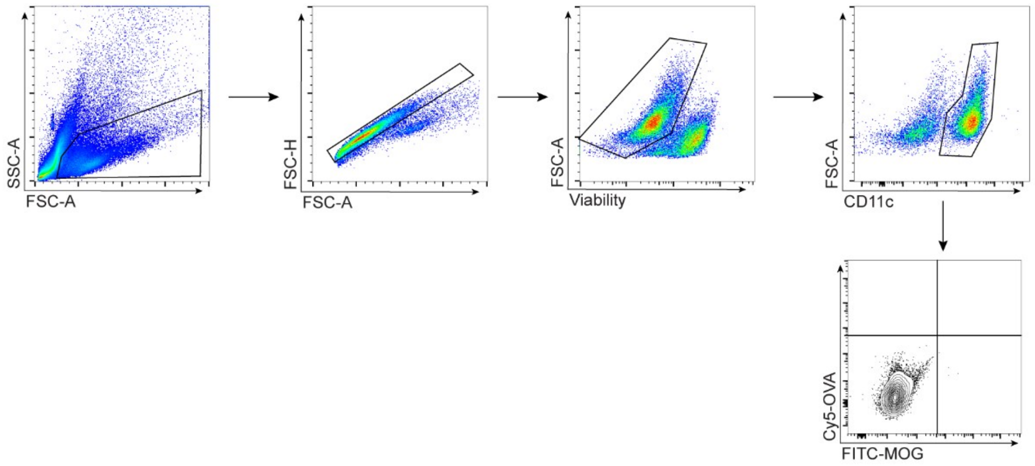

Figure S7. Gating scheme for CD11c+ DCs. DCs are gated on FITC vs. Cy5 to identify fluorescently-labeled MP uptake.

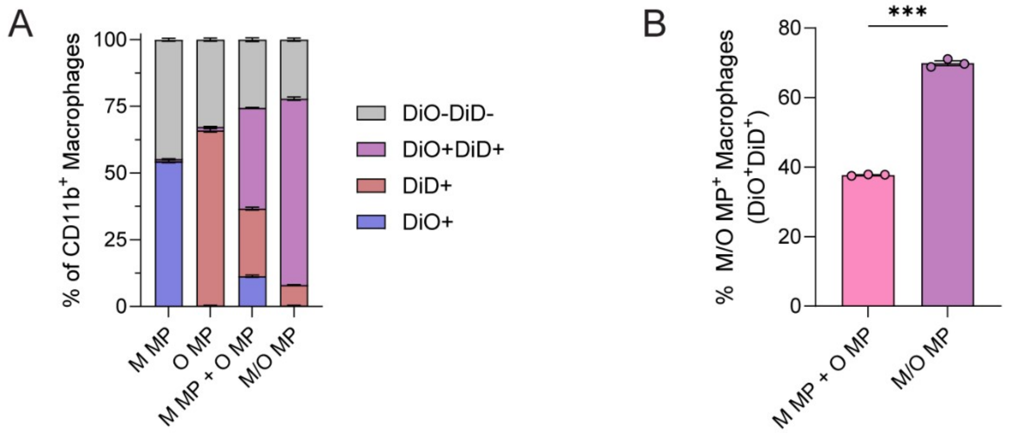

Figure S8. (A) Quantification of frequency of macrophages without MPs (no fluorescent signal) or with MOG MPs (DiO$^+$), OVA MPs (DiD$^+$), mixed MPs (DiO$^+$/DiD$^+$) or co-loaded MPs (DiO$^+$/DiD$^+$). (B). Quantification of macrophages frequency that contains MPs with both peptides. Data is representative of two or more experiments. ***P < 0.001 determined with two-tailed Welch's t-test.

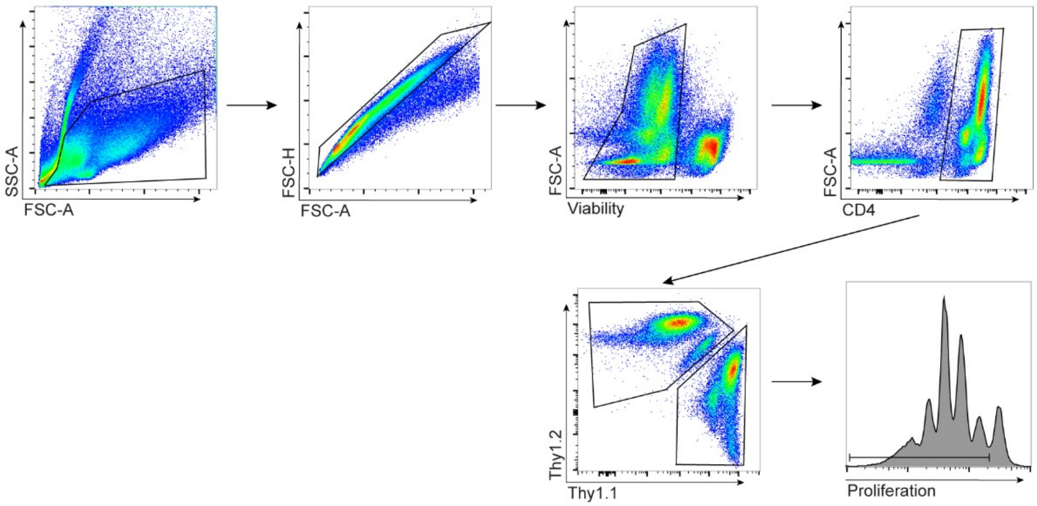

Figure S9. Gating scheme for *in vitro* T cell proliferation. CD4$^+$ T cells are gated on Thy1.1 vs Thy1.2 to identify 2D2 (Thy1.1$^+$) and OT-II (Thy1.2$^+$) positive cells. E Fluor 450 proliferation was gated on all peaks beyond the first (G0, representing undivided cells), using undivided controls.

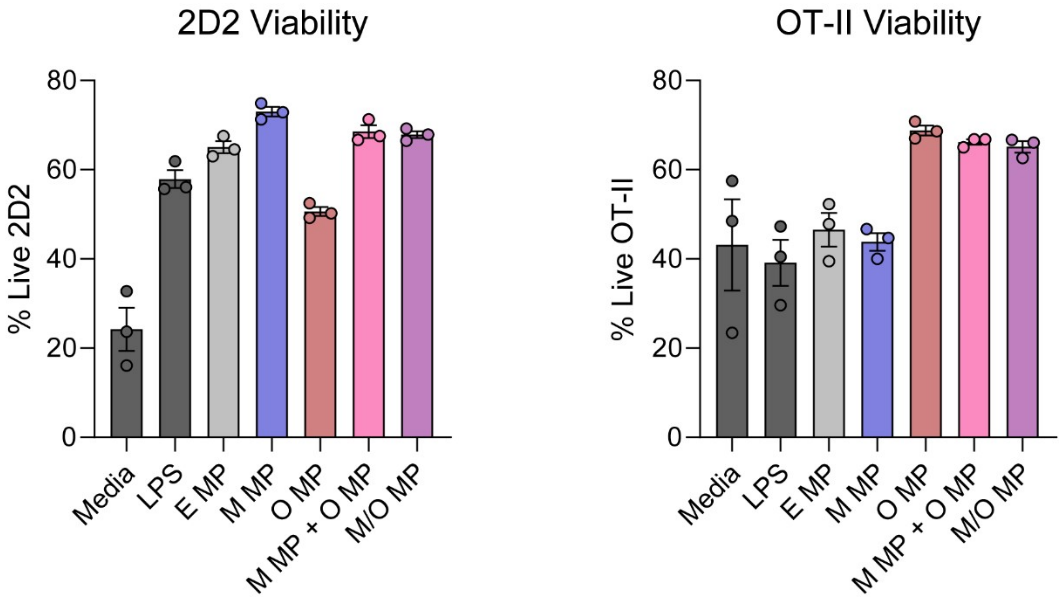

Figure S10. Viability of 2D2 and OT-II T cells after MP treatment.

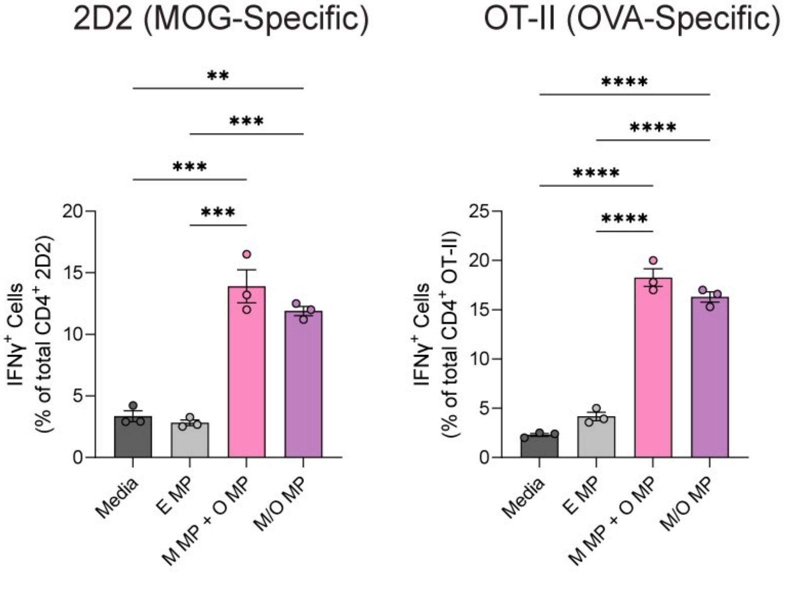

Figure S11. IFN-γ production of restimulated, MP-treated, antigen-specific $CD4^+$ T cells from each indicated strain.

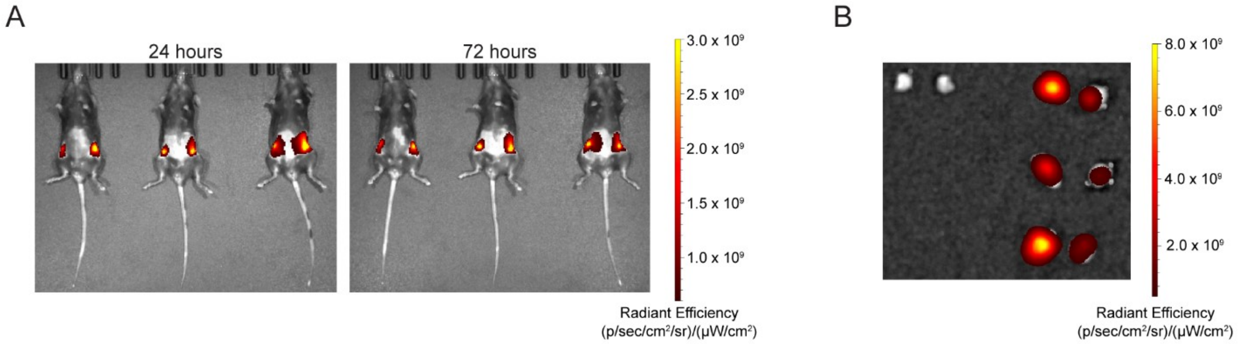

Figure S12. IVIS imaging over 72 hours demonstrating MP localization to the injected LN after iLN treatment. (A) *In vivo* imaging of mice 24 and 72 hours post iLN injection with fluorescent MPs. (B) Image of excised LNs after iLN injection.

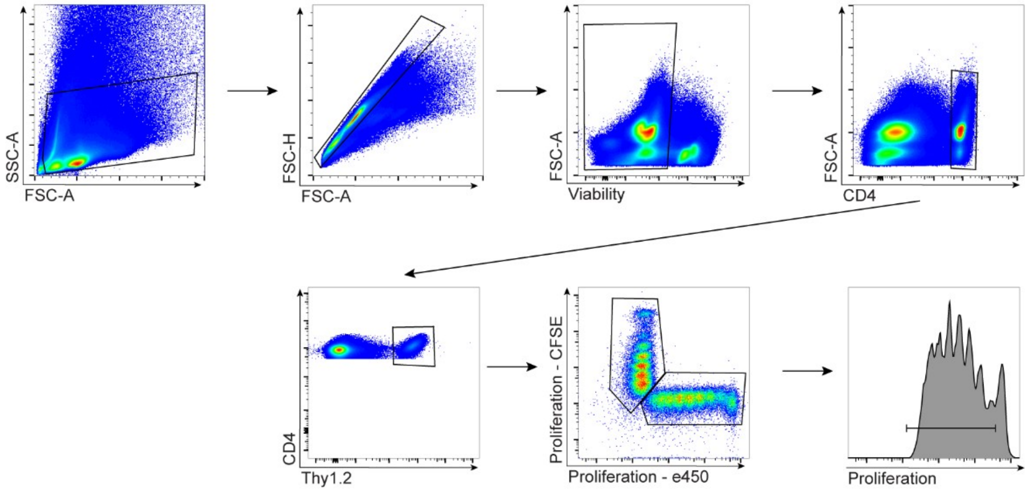

Figure S13. Gating scheme for *in vivo* T cell proliferation. All transferred cells are CD4⁺Thy1.2⁺. 2D2 (e450) and OT-II (CFSE) cells were identified using labeled proliferation dyes. Proliferation was gated on all peaks beyond the first (G0), using undivided controls.

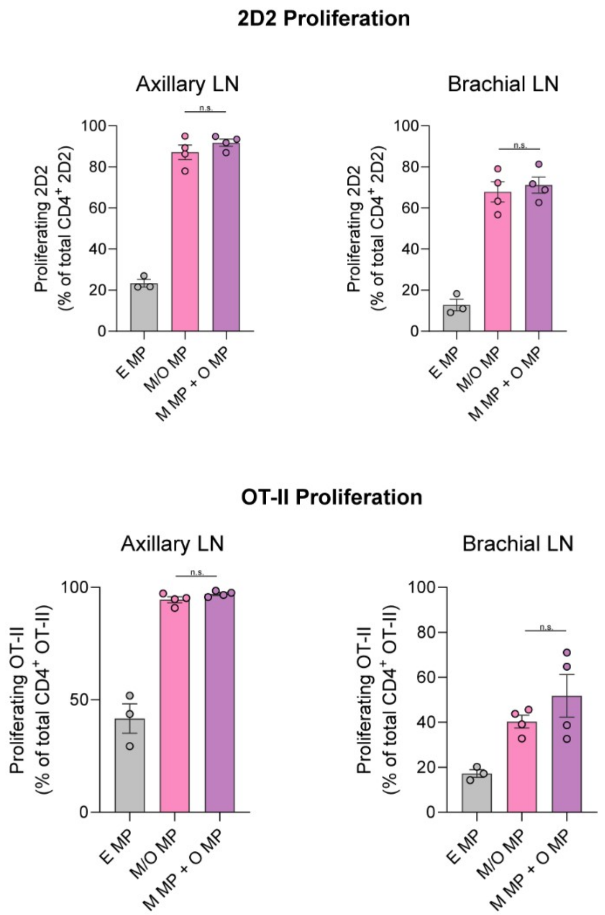

Figure S14. Frequency of proliferating recovered 2D2 or OT-II cells in axillary and brachial LNs.

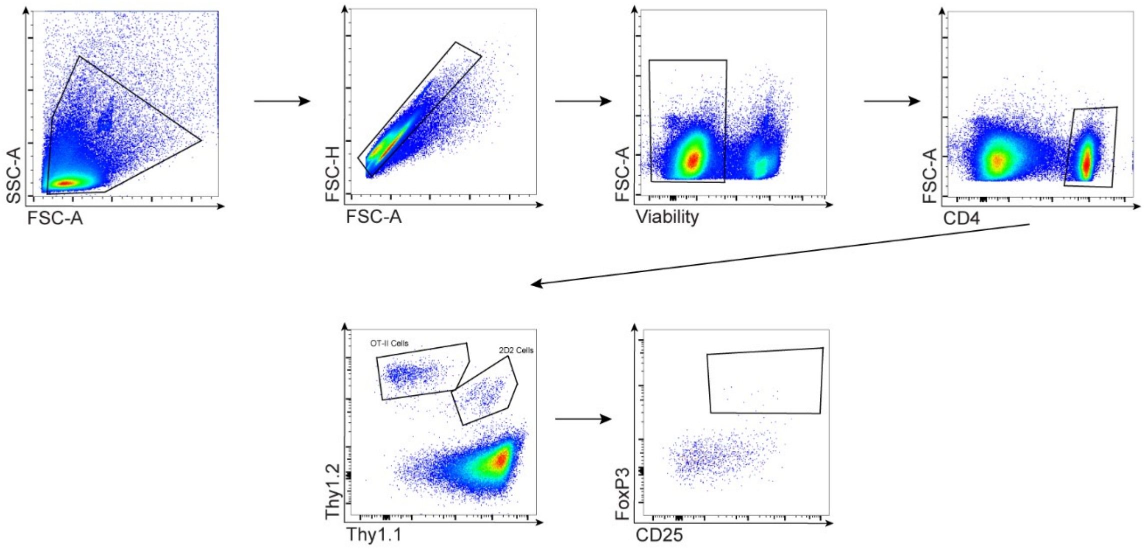

Figure S15. Gating scheme for *in vivo* T cell phenotype. 2D2 ($Thy1.1^{+}Thy1.2^{+}$) and OT-II ($Thy1.2^{+}$) cells were obtained by gating $CD4^{+}$ T cells using Thy1.1 vs. Thy1.2. T_{reg} were identified as $CD25^{+}FoxP3^{+}$.

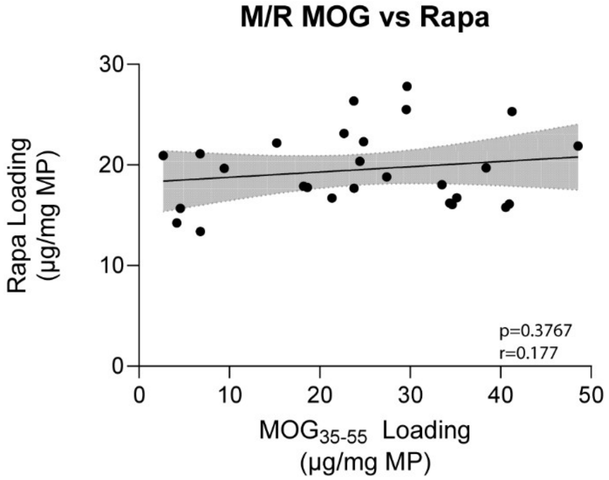

Figure S16. Correlation between $MOG_{35\text{-}55}$ peptide loading and rapa loading in M/R MPs.

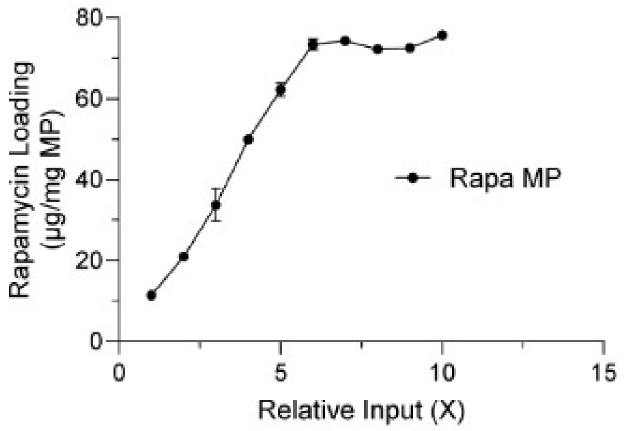

Figure S17. Loading curve of rapa in rapa MPs.

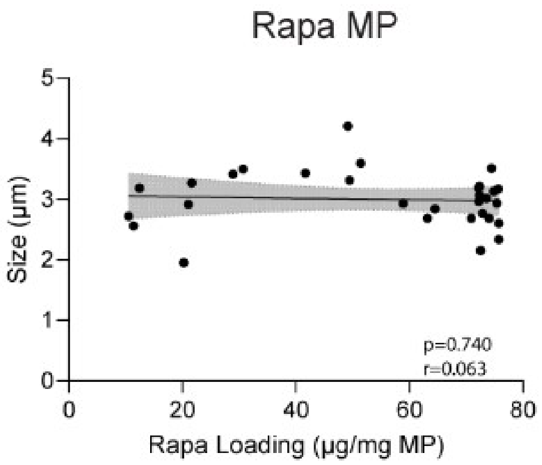

Figure S18. Correlation between size and rapa loading in rapa MPs.
